# Mitochondrial Dysfunction and Calcium Dysregulation in Leigh Syndrome Induced Pluripotent Stem Cell Derived Neurons

**DOI:** 10.3390/ijms21093191

**Published:** 2020-04-30

**Authors:** Teresa Galera-Monge, Francisco Zurita-Díaz, Isaac Canals, Marita Grønning Hansen, Laura Rufián-Vázquez, Johannes K. Ehinger, Eskil Elmér, Miguel A. Martin, Rafael Garesse, Henrik Ahlenius, M. Esther Gallardo

**Affiliations:** 1 Departamento de Bioquímica, Facultad de Medicina, Universidad Autónoma de Madrid, 28029 Madrid, Spain; tgaleramonge@gmail.com (T.G.-M.); frazurdia@gmail.com (F.Z.-D.); rafael.garesse@uam.es (R.G.); 2 Departamento de Modelos Experimentales de Enfermedades Humanas, Instituto de Investigaciones Biomédicas “Alberto Sols” UAM-CSIC, 28029 Madrid, Spain; 3 Centro de Investigación Biomédica en Red (CIBERER), 28029 Madrid, Spain; laurarufian@gmail.com (L.R.-V.); mamcasanueva.imas12@h12o.es (M.A.M.); 4 Instituto de Investigación Sanitaria Hospital 12 de Octubre (i + 12), 28041 Madrid, Spain; 5 Department of Clinical Sciences, Neurology, Lund Stem Cell Center, Lund University, 221 00 Lund, Sweden; isaac.canals@med.lu.se (I.C.); marita.gronning_hansen@med.lu.se (M.G.H.); 6 Laboratorio de enfermedades mitocondriales y Neurometabólicas, Hospital 12 de Octubre, 28041 Madrid, Spain; 7 Mitochondrial Medicine, Department of Clinical Sciences Lund, Faculty of Medicine, Lund University, BMC A13, 221 84 Lund, Sweden; johannes.ehinger@med.lu.se (J.K.E.); eskil.elmer@med.lu.se (E.E.); 8 Grupo de Investigación Traslacional con células iPS. Instituto de Investigación Sanitaria Hospital 12 de Octubre (i + 12), 28041 Madrid, Spain

**Keywords:** Leigh syndrome, mitochondrial disorder, iPSC, NSC, neuron, disease modeling, mtDNA

## Abstract

Leigh syndrome (LS) is the most frequent infantile mitochondrial disorder (MD) and is characterized by neurodegeneration and astrogliosis in the basal ganglia or the brain stem. At present, there is no cure or treatment for this disease, partly due to scarcity of LS models. Current models generally fail to recapitulate important traits of the disease. Therefore, there is an urgent need to develop new human in vitro models. Establishment of induced pluripotent stem cells (iPSCs) followed by differentiation into neurons is a powerful tool to obtain an in vitro model for LS. Here, we describe the generation and characterization of iPSCs, neural stem cells (NSCs) and iPSC-derived neurons harboring the mtDNA mutation m.13513G>A in heteroplasmy. We have performed mitochondrial characterization, analysis of electrophysiological properties and calcium imaging of LS neurons. Here, we show a clearly compromised oxidative phosphorylation (OXPHOS) function in LS patient neurons. This is also the first report of electrophysiological studies performed on iPSC-derived neurons harboring an mtDNA mutation, which revealed that, in spite of having identical electrical properties, diseased neurons manifested mitochondrial dysfunction together with a diminished calcium buffering capacity. This could lead to an overload of cytoplasmic calcium concentration and the consequent cell death observed in patients. Importantly, our results highlight the importance of calcium homeostasis in LS pathology.

## 1. Introduction

Leigh syndrome (LS) is the most frequent infantile mitochondrial disorder (MD) with a prevalence of 1 in 40,000 births [1,2,3]. Forty-five percent of LS patients die before reaching 20 years of age, most of them by respiratory failure. However, there are several complications that may further increase morbidity and mortality, such as refractory seizures and cardiovascular deterioration [2]. The common feature of these patients is the presence of bilateral symmetric necrotic areas in the basal ganglia or the brain stem, which correspond with regions of demyelination, neuronal death and astrogliosis [1,2]. However, LS is characterized by a prominent clinical and genetic variability. More than 75 genes have been associated to LS [3], all of them involved in mitochondrial energy production [3].

Mitochondria are cellular organelles considered to be the powerhouse of the cell because of their participation in cellular energy production through a process known as oxidative phosphorylation (OXPHOS) [4]. The OXPHOS process is carried out by five multiheteromeric complexes located in the inner mitochondrial membrane and collectively termed the respiratory chain (RC) [5]. Moreover, mitochondria are also involved in other pivotal processes such as reactive oxygen species (ROS) production, apoptosis and calcium homeostasis, whose role in pathology is being increasingly recognized. Given the crucial role of mitochondria for functionality of neuronal cells, it is not surprising that diseases affecting mitochondria result in neurological conditions such as LS [6].

Mitochondria possess their own DNA [7] and the human mitochondrial DNA (mtDNA) is a double-stranded circular molecule of 16.5 kb that encodes 13 subunits of the OXPHOS complexes as well as two rRNAs and 22 tRNAs [7]. Hundreds to thousands of copies of mtDNA are present per cell [5,8], allowing the possibility of coexistence of healthy and pathogenic mtDNA molecules, a phenomenon called heteroplasmy. Genetically, LS is highly heterogeneous; to date, a broad variety of causative mutations have been described in nuclear- and mitochondrial-encoded genes involved in energy metabolism. mtDNA mutations are responsible for 10–20% of LS cases and, more specifically, mutations in genes affecting complex I of the respiratory chain have been a well-recognized cause of LS. Among them, the m.13513G>A mutation located in the *MT-ND5* gene is a frequent cause of LS [9]. In spite of the advances in the molecular diagnosis of LS, the molecular pathogenesis of this disease remains poorly understood due, in part, to the lack of suitable disease models.

For that reason, generation of induced pluripotent stem cells (iPSCs) and differentiation into the affected tissue could be an interesting approach for modeling LS [10]. Several studies have used iPSC technology to generate in vitro models of LS harboring the mutation m.8993T>G in the *MT-ATP6* gene [11,12]. These models recapitulate the mitochondrial dysfunction in muscle [11] or in neurons, the principally affected cell type [12]. Moreover, this model recapitulated the typical neurodegeneration in brains of LS patients [12]. The m.13513G>A mutation in the *MT-ND5* gene is responsible for mitochondrial myopathy, encephalopathy, lactic acidosis and stroke (MELAS) and LS. Until now, one report has described the behavior of heteroplasmy during reprogramming and extended culture of iPSCs harboring this mutation in association with MELAS syndrome, but neuronal characterization is missing. Here, we generate iPSC-derived neurons from a described patient suffering LS caused by m.13513G>A mutation in heteroplasmy [9] and explore the mechanisms by which this mutation could cause the disease. Although LS iPSC-derived neurons were electrophysiologically normal, they manifested a decreased respiration and a diminished calcium buffering capacity. The slower removal of cytoplasmic calcium could lead to an overload and the consequent neuronal death observed in patients.

## 2. Results

### 2.1. Diminished Respiration in LS Fibroblasts Caused by a Decrease of Mitochondrial Mass

**Figure 1:**
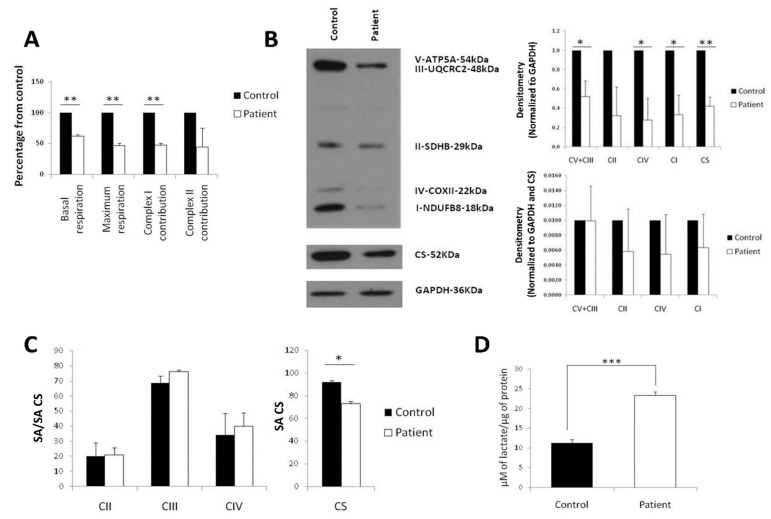
Decreased mitochondrial mass and respiration in Leigh syndrome (LS) fibroblasts. (**A**) Oxygen consumption measured in Oroboros Oxygraph-2k. All data are displayed as a percentage of control. (**B**) Western blot assay against mitoprofile, citrate synthase (CS) and GAPDH (left). Quantification of the Western blot, normalized with GAPDH as a marker of the total protein (right, top panel) or GAPDH and citrate synthase (CS) as a marker of mitochondrial mass (right, bottom panel). All data are displayed as a percentage of control. (**C**) Spectrophotometric measurements of the activity of electron transfer chain (ETC) complexes (left) and citrate synthase (CS) (right); SA: specific activity. (**D**) Extracellular lactate production normalized by total protein. (* *p*-value < 0.05; ** *p*-value < 0.01; *** *p*-value < 0.001)

As a first step to test mitochondrial function in human LS cells, we subjected LS fibroblasts harboring the *MT-ND5* m.13513G>A mutation, with a mutant load of 55%, and control fibroblasts to high-resolution respirometry on an Oroboros Oxygraph-2k. Measurements revealed that basal respiration (Cr-ROX), maximal respiratory capacity (CrU-ROX) and complex I contribution to respiration, CrU-(CRot-ROX), were lower in patient as compared to control fibroblasts (Figure 1A). However, albeit lowered to a similar extent, no significant difference was detected in complex II contribution to respiration (CRot-ROX) (Figure 1A). To further understand if the respiratory deficiency was provoked by diminished mitochondrial mass or quantity of RC complexes, a Western blot against subunits of complexes I-V and citrate synthase (CS) as a marker of mitochondrial mass was performed. A diminished mitochondrial mass was indeed observed in Leigh fibroblasts, accounting for the defect in complexes I, III+V and IV (Figure 1B). A defect in complexes I, III+V and IV was detected when normalizing with GAPDH but not when normalizing with both CS and GAPDH (Figure 1B). Moreover, analysis of enzymatic activities of respiratory complexes highlighted a normal function of complexes II, III and IV (CS normalized) but a decreased activity of CS in LS fibroblasts (Figure 1C), supporting a decrease in mitochondrial mass as the main contribution to the lower levels of respiration. Finally, extracellular lactate measurements demonstrated that LS fibroblasts produced more than twice the lactate generated by control fibroblasts (Figure 1D), which is consistent with the decreased respiration of LS fibroblasts.

### 2.2. Generation of the Control iPSc Line N44SV.1

Since LS models are scarce, generation of LS iPSCs and their differentiation into neural lineages would allow to shed light on the pathological mechanisms causing this disease.

To model LS, we used a previously obtained iPSC line, LND554SV.4, with the mutation m.13513G>A at a percentage of 45% [13]. This line was derived from the same patient fibroblasts where we detected diminished respiration. As a control, we generated the iPSC line (N44SV.1) from normal human dermal fibroblasts (NHDFs) using Sendai virus. We could confirm by sequencing that it, in contrast to the LS iPSC line, lacked the m.13513G>A mutation in the mtDNA (See Supplementary Figure S1A). Moreover, the N44SV.1 line displayed hES-like colonies positive for the pluripotency marker alkaline phosphatase (see Supplementary Figure S1B,C). N44SV.1 also showed high levels of mRNAs of pluripotency-related genes *OCT4*, *SOX2*, *CRIPTO*, *NANOG* and *REX1* in comparison with the original fibroblasts (see Supplementary Figure S1D). At the protein level, we detected by immunofluorescence the presence of pluripotent surface proteins SSEA3, SSEA4, Tra-1-81 and Tra-1-60 and the pluripotent transcription factors OCT4, SOX2 and NANOG (see Supplementary Figure S1E). The ability to differentiate into the three germ layers was tested using an embryoid body (EB)-based methodology. Endoderm (positive for AFP), mesoderm (positive for SMA) and neuroectoderm cells (positive for TUJ1) were observed (see Supplementary Figure S1F). Moreover, N44SV.1 presented a complete clearance of the Sendai viruses used for inducing reprogramming (see Supplementary Figure S1G) and a normal karyotype (see Supplementary Figure S1H). Finally, DNA fingerprinting analysis revealed genetic identity of N44SV.1 with control NHDFs (see Supplementary Figure S1I). Importantly, we did not detect any difference in the reprogramming process or in the pluripotency markers between LND554SV.4 [13] and the control iPSC line, N44SV.1.

### 2.3. LS iPSCs Manifest a Decreased Basal Respiration and a Combined RC Deficiency

**Figure 2:**
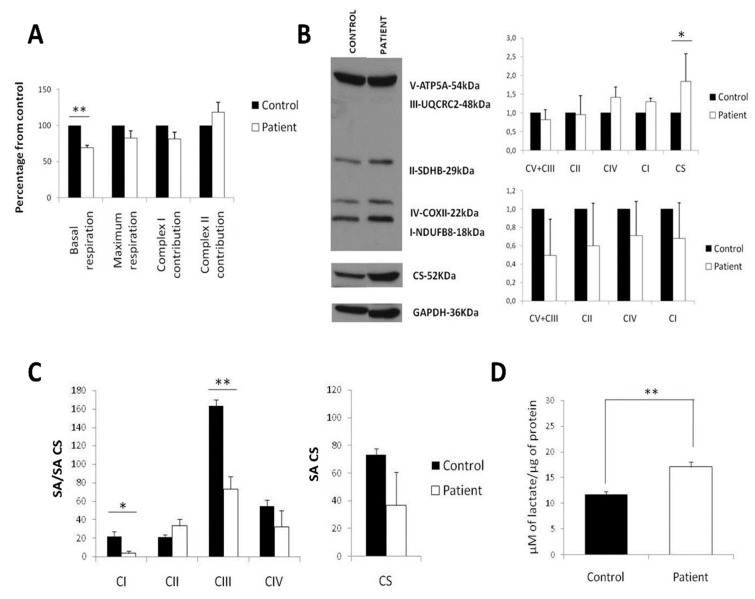
LS induced pluripotent stem cells (iPSCs) manifest a decreased basal respiration and a combined respiratory chain (RC) deficiency. (**A**) Oxygen consumption measured in Oroboros Oxygraph-2k. All data are displayed as a percentage of control. (**B**) Western blot assay against mitoprofile, citrate synthase (CS) and GAPDH (left). Quantification of the Western blot, normalized with GAPDH as a marker of the total protein (right, top panel) or GAPDH and citrate synthase (CS) as a marker of mitochondrial mass (right, bottom panel). All data are displayed as a percentage of control. (**C**) Spectrophotometric measurements of the activity of ETC complexes (left) and citrate synthase (CS) (right); SA: specific activity. (**D**) Extracellular lactate production normalized by total protein. (* *p*-value < 0.05; ** *p*-value < 0.01; *** *p*-value < 0.001)

Afterwards, we analyzed metabolic function of LS iPSCs by performing a mitochondrial characterization. Oxygen consumption measurements revealed that basal respiration (Cr-ROX) was diminished in LS iPSCs, while maximum respiration (CrU-ROX) and complex I contribution, CrU-(CRot-ROX), were not significantly decreased. Complex II contribution (CRot-ROX) was unaltered as compared to control iPSCs (Figure 2A). Except for a significant increase in CS, analysis of mitochondrial mass and protein content of RC complexes by Western blot demonstrated no major differences between the patient and the control (Figure 2B). However, activity measurements showed a prominent defect in the activity of complexes I and III of LS iPSC (Figure 2C). Similar to fibroblasts, lactate levels were increased in LS iPSCs as compared to control iPSCs (Figure 2D).

These findings show that hyperlactacidemia (a molecular marker of mitochondrial dysfunction commonly found in LS) can be detected as increased lactate levels in both fibroblasts and iPSCs derived from LS patient fibroblasts.

### 2.4. Similar Proliferation and Differentiation Capacity of LS iPSC-Derived NSCs

**Figure 3:**
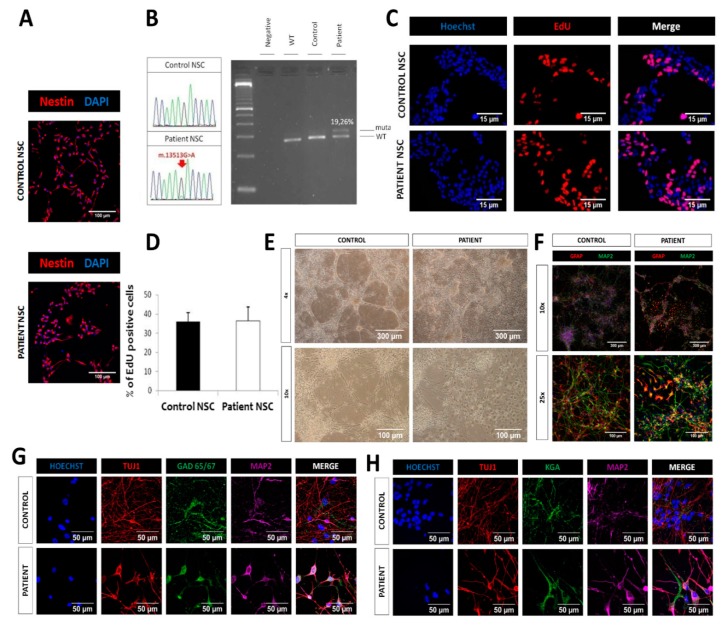
LS neural stem cells (NSCs) manifested similar proliferative and differentiation capacity. (**A**) Immunofluorescence analysis of the neural stem cell marker nestin, manifesting an efficient generation of NSCs from iPSCs; scale bar: 100 µm. (**B**) Electropherograms showing the mutation m.13513G>A in patient NSCs and its absence in control NSCs (left) and heteroplasmy levels of m.13513G>A mutation by RFLP followed by Agilent quantification. (**C**) Proliferation assay of NSCs with the thymidine analogue 5-ethynyl-2’-deoxyuridine (EdU). Scale bar: 15 µm. (**D**) Quantification of EdU (percentage of EdU+/Hoechst+). (**E**) Bright field images (4× and 10×) of neural populations obtained after differentiation of NSCs. (**F**) Immunofluorescence analysis of MAP2, a marker of mature neurons, and GFAP, a marker of astrocytes, in the neural populations obtained after differentiation of NSCs in N2B27 for 3 weeks (**G**–**H**) Immunofluorescence analysis of the GABAergic marker GAD 65/67 and glutamatergic marker KGA together with neuronal markers (Tuj1 and MAP2).

In order to assess pathology in cells more relevant to the disease, we derived neural stem cells (NSCs) from control and LS iPSCs. NSCs were efficiently derived from both patient and control iPSCs and stained positive for the NSC marker nestin (Figure 3A). The m.13513G>A mutation was retained in patient NSCs at a percentage of 19.26% while absent in control NSCs (Figure 3B). No differences were observed in the percentages of EdU+ cells between control and patient NSCs (Figure 3C,D), indicating a similar proliferative rate. Differentiation capacity was tested at 3 weeks of culture in differentiation media. At that time point, control NSCs had developed a network of neuronal cells with clustering of somas and interconnecting neurites (Figure 3E). The tendency to cluster was less strong in patient NSCs, which showed a less organized network. Immunocytochemistry for the neuronal marker MAP2 and the astrocytic marker GFAP revealed that most of the cells, both in control and patient, were MAP2+, and only a few of them were GFAP+ (Figure 3F). In order to study the subtype of neurons present in the culture, we allowed NSCs to differentiate for 6 weeks and analyzed by immunofluorescence the presence of the glutamatergic marker KGA and the GABAergic marker GAD65/67. Both control and patient NSCs generated MAP2+ or TUJ1+ cells, which co-stained with KGA or GAD65/67, without any obvious differences between groups (Figure 3G,H), proving the presence of both glutamatergic and GABAergic neurons in our cultures.

### 2.5. Cell death and Complex I deficiency in LS iPSC-Derived Neurons

**Figure 4:**
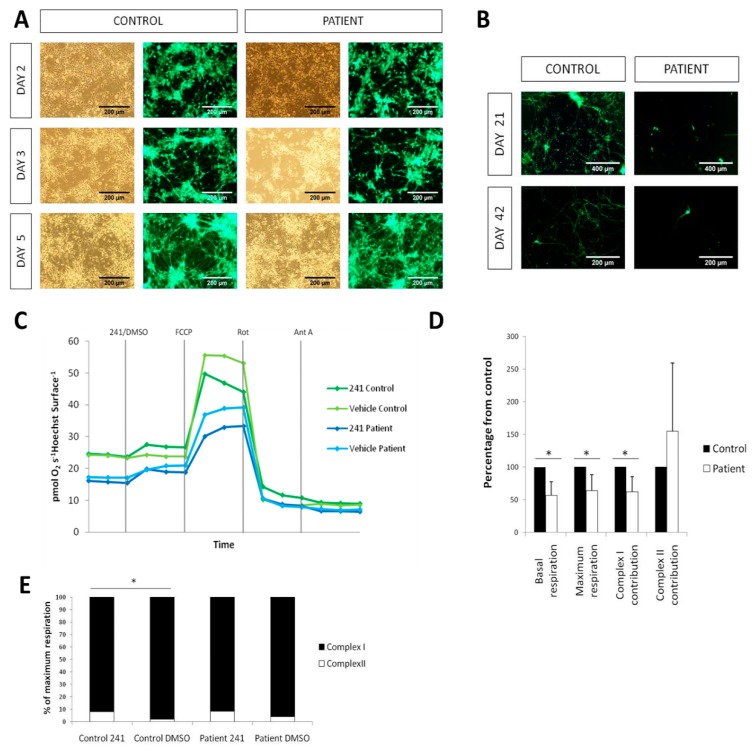
Respiratory defect and neurodegeneration of patient iPSC-derived neurons. (**A**) iN generation from iPSCs using lentiviral vectors for NgN2, rtTA and GFP showing no alterations in derivation of iNs from the patient. (**B**) iNs co-cultured with mouse astrocytes showing a marked neurodegeneration in the patient in comparison with the control both at days 21 and 42. (**C**) Oxygen consumption plots of the different treatments (Control/Patient and NV241/DMSO). (**D**) Quantification of oxygen consumption measured in a Seahorse XFe96 Analyzer. All data are normalized with the control. (**E**) Quantification of the contributions of complexes I and II to the maximum respiration, in percentages. (* *p*-value < 0.05)

In order to specifically analyze neuronal properties, we used a previously published protocol [14] to induce a pure population of neurons (iPSC-iNs) from control and LS iPSCs. To analyze appearance of neuronal networks, sparse cytoplasmic lentiviral GFP labeling was used. Initially, neuronal networks derived from control and patient iPSCs appeared similar (Figure 4A). However, after prolonged culture on mouse astrocytes, pronounced neuronal death was observed in cultures from patient iPSCs at day 21; this was further aggravated at day 42 (Figure 4B).

Measurement of oxygen consumption was performed on a Seahorse Analyzer directly on attached iNs without replating to avoid cell death. A complex I deficiency was detected in patient iNs evidenced by a decrease of the basal respiration (Cr-ROX), maximum respiratory capacity (CrU-ROX) and complex I contribution, CrU-(CRot-ROX), to maximal respiratory capacity (Figure 4C,D). Complex II contribution (CRot-ROX) was not different in patient iNs as compared to control (Figure 4C,D). Treatment with the cell-permeable succinate prodrug NV241 resulted in a similar increase in routine respiration in both patient and control iNs, however the response in patient iNs vehicle (DMSO) control also displayed increased respiration which rendered the NV241 response data in the patient iNs inconclusive (Figure 4C). Further, the treatment with the succinate prodrug increased complex II contribution to maximal uncoupled respiration in control iNs (Figure 4E). In patient iNs, the succinate prodrug induced a similar level of complex II contribution to maximal uncoupled respiration, but the difference to vehicle (DMSO) control did not reach significance (Figure 4E).

### 2.6. LS iPSC-Derived Neurons are Functional

**Figure 5:**
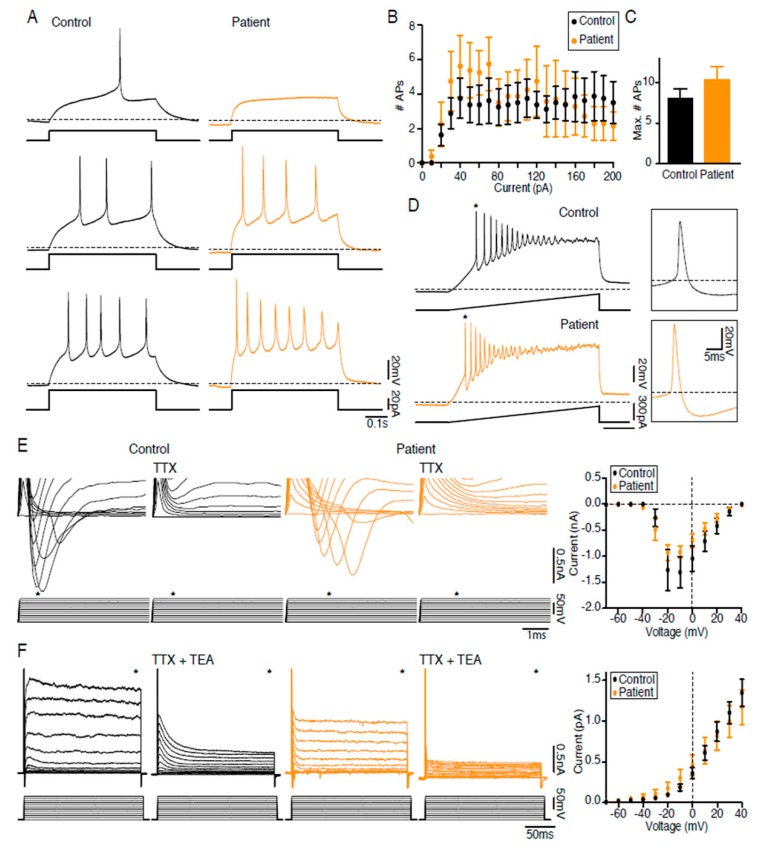
LS iPSC-derived neurons are electrophysiologically functional. (**A**) Neurons derived from control (black) and patient (orange) neural stem cells were able to generate action potentials (APs) upon current injection. (**B**) Graph showing the injected current versus the number of APs elicited (*n* = 8). (**C**) Bar diagram of the maximal number of APs induced (*n* = 8, n.s.). (**D**) Voltage traces show that current injection (ramp from 0–300 pA) induces trains of APs; * denotes the expanded AP17. (**E**,**F**) Left: Current traces of the fast inward current peak (**E**) and the sustained outward current (**F**) activated by step depolarizations from a holding potential of −70 mV in the absence and presence of 1 mM TTX (**E**) or 1 mM TTX and 10 mM TEA (**F**); * denotes the fast inward current peak (**E**) and the sustained outward current (**F**). Right: Voltage–current plot of the inward current peak (**E**) and sustained outward current (**F**).

In order to test whether the alteration in mitochondrial function has an effect on neuronal function, electrophysiological properties of LS neurons obtained, after a differentiation period of six weeks, were analyzed using whole-cell patch clamp. Neurons differentiated from both control and patient iPSC-derived NSCs were able to fire action potentials (APs) upon current injection (Figure 5A), and the number of elicited APs in each current step injected was not different between groups (Figure 5B). Moreover, the maximal number of elicited APs was similar (Figure 5C), and the application of ramps of currents triggered trains of APs in patient neurons in the same way as in controls (Figure 5D). Furthermore, both control and patient neurons had depolarizing inward Na^+^ currents blocked by TTX (Figure 5E) and repolarizing outward K^+^ currents blocked by TTX + TEA (Figure 5F). In conclusion, no abnormalities were detected in intrinsic properties or AP characteristics of patient neurons as compared to control.

### 2.7. Disturbed Calcium Regulation in LS iPSC-Derived Neurons

**Figure 6:**
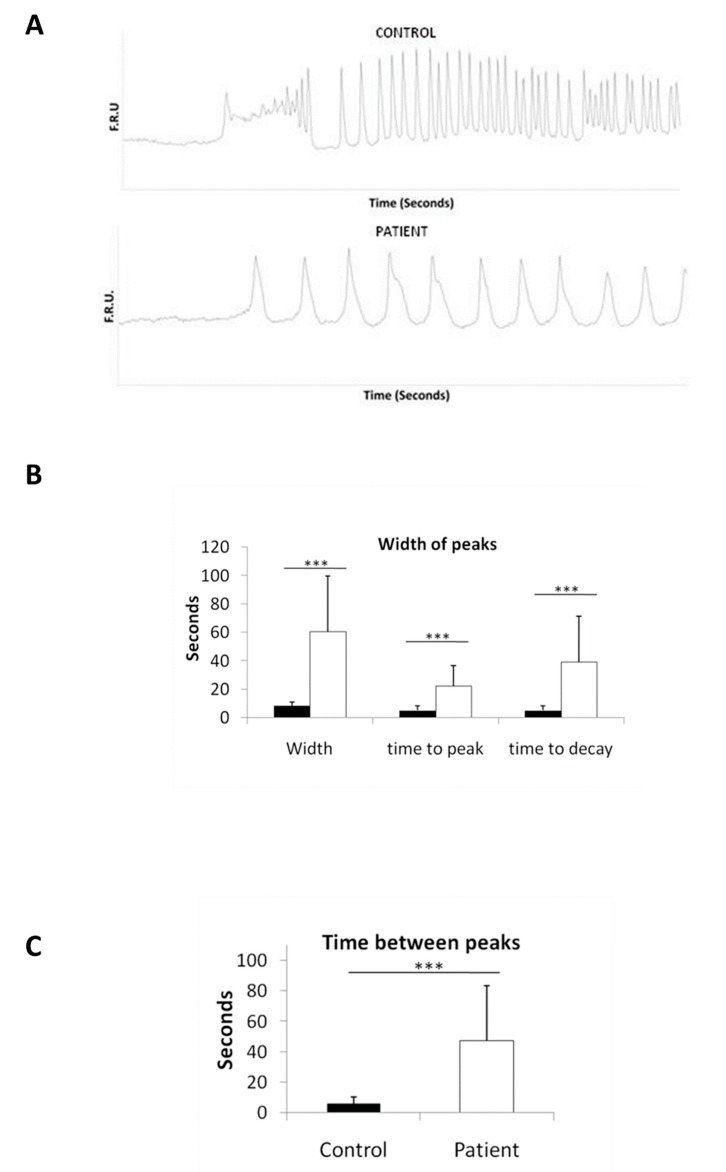
Calcium dysregulation in LS iPSC-derived neurons. (**A**) Representative plots of calcium imaging displaying a different response to KCl in LS iPSC-derived neurons. FRU: fluorescence relative units. (**B**) Quantification of the width of the peaks (from basal to basal), time to peak (from basal to peak) and time to decay (from peak to basal). (**C**) Quantification of the time between peaks. (*** *p*-value < 0.001)

Given the role of the mitochondria in regulating intracellular calcium we wanted to assess calcium dynamics in LS neurons. Intracellular calcium concentrations were analyzed by live cell calcium imaging in NSC-derived neurons, after 6 weeks of differentiation. KCl was added in order to stimulate neuronal activity, and the number of evoked cells (responding to KCl) was drastically diminished in patient neurons as compared to controls (see Supplementary Video S1 (Control) and Supplementary Video S2 (Patient)). Moreover, patient evoked cells showed a very different response, compared to controls, with increase in the width of peaks (time from basal to basal level) and time between peaks (Figure 6A). Quantification confirmed the increase in the width of peaks (Figure 6B), which could be explained by a slower increase in cytoplasmic Ca^2+^ upon a depolarizing stimulus or a decrease in the calcium buffering capacity. In order to understand the specific step affected, peaks were measured as time to peak (from basal to maximum level) and time to decay (from maximum to basal level). Although both parameters were increased in patient cells, time to decay was more affected, indicating a Ca^2+^ buffering defect (Figure 6B). Time between peaks was also assessed and showed an increase of the refractory period in patient neurons (Figure 6C). All these results together indicate a dysregulation in calcium homeostasis caused by the m.13513G>A mutation.

## 3. Discussion

All mammalian cells produce ATP by glycolysis and OXPHOS. The balance between these processes is different in each cell type [15], and its disturbance can cause disease. Here, we show a clearly compromised OXPHOS function in LS patient fibroblasts and neurons; however only basal respiration was altered in patient iPSCs. This indicates that the defect in iPSC lines is less evident than in fibroblasts or neurons, probably due to the main dependence of iPSCs on glycolysis [6,15,16]. This conclusion is supported by the low levels of oxygen consumed by iPSCs: basal respiration in control iPSCs was approximately half and maximum respiration one-third of that of control fibroblasts.

Theoretically, a defect in complex I of the respiratory chain could be bypassed by an increase in the electron flow through complex II that could restore ∆Ψmit and ATP levels. In this regard, succinate fuels complex II but it cannot cross biological membranes and does not reach mitochondria. Recently, several cell-permeable succinate prodrugs have been described [17]. As LS patient neurons manifested a decreased complex I respiration, we attempted to rescue that phenotype using one of the succinate prodrugs, NV241, described by Ehinger et al. [17]. Administration of the succinate prodrug increased both routine (basal) respiration and the complex II contribution to maximal respiration to similar levels in patient and control iPSC-derived neurons. However, patient neurons responded with increased respiration, possibly uncoupling, to the drug vehicle (DMSO), which rendered the drug response data inconclusive for the patient iPSC-derived neurons. For the control neurons, the stimulating effects of complex II on respiration were significant. In the initial publication describing the cell-permeable succinate prodrugs, respiration and spare respiratory capacity were increased by prodrug administration in LS patient fibroblasts with a recessive *NDUFS2* mutation.

The question remains as to why iPSCs with their low dependence on OXPHOS manifest a combined RC defect with decreased activities of complexes I and III, while fibroblasts, which rely on mitochondrial OXPHOS more than iPSCs [16], do not show a diminished activity of complexes III and IV similar to muscle. Due to technical problems it was not possible to measure complex I deficiency. Therefore, we cannot discard the possibility of an isolated complex I deficiency in fibroblasts. Sequencing of mitochondrial complex I related genes, however, ruled out the possibility that control fibroblasts had mutations that were not previously detected. One possible explanation for normal specific activity of complexes III and IV in fibroblasts is that the alteration of the ETC function in patient fibroblasts could lead to a decreased ∆Ψmit, which in turn could induce an increase in the selective elimination of abnormal mitochondria through mitophagy and mask the underlying defect when enzymatic activities are measured. We do observe, as the main finding in patient fibroblasts, diminished mitochondrial mass; considering that mitophagy is the core mechanism of mitochondrial quality and quantity control [18], it is reasonable to think that mitophagy could account for the decrease in mitochondrial mass. This compensatory effect has already been described in other fibroblasts with OXPHOS mutations [19,20,21,22], and its protective role during pathogenesis is recognized [22]. However, in one of these studies, no increase of mitophagy in fibroblasts harboring the m.13513G>A mutation was observed [22]. We therefore cannot rule out the possibility that the observed reduction in protein levels and activity in CS could be the consequence of a decreased mitochondrial biogenesis or an increase in the random elimination of mitochondria by bulk macroautophagy. This alternative explanation, in which no selection against abnormal mitochondria occurs, would not, however, explain the normal activity found in complexes III and IV in the fibroblasts.

In contrast to what happen in fibroblasts, iPSCs, similar to cancer cells, are considered to mainly rely on glycolysis [6,15,16]. In theory, the low OXPHOS function in iPSCs would lead both in controls and patients to a decrease of ∆Ψmit and the consequent activation of mitophagy [18,23]. It has been demonstrated that iPSCs display higher ∆Ψmit than differentiated cells [24,25], even using hydrolase activity of ATP synthase to maintain ∆Ψmit [26]. The maintenance of normal ∆Ψmit would prevent mitophagy and the compensatory effect on patient iPSCs; as a consequence, no abnormal complexes would be removed, and a compromised activity of complexes I and III would be detected even when the defect is not evident at physiological conditions.

For disease modeling, it is important to recapitulate the principal pathological features of the disease. LS is characterized by the presence of bilateral symmetric necrotic areas in the basal ganglia or the brain stem which correspond with regions of demyelination, neuronal death and astrocytic gliosis [1,2]. Diseased neurons manifested a compromised respiratory capacity and evident neuronal death after being replated on mouse astrocytes. These results indicate that this in vitro model, at least in part, recapitulates the in vivo phenotype of LS. Moreover, increased lactate levels in blood or cerebrospinal fluid are common criteria in the diagnosis of MD. Here, both cell types analyzed (fibroblasts and iPSCs) manifest elevated production of lactate, independently of the main pathway used by the cell type to generate energy.

Patch clamp recordings revealed normal electrophysiological function of individual patient neurons, indicating that mtDNA mutation m.13513G>A does not impair Na^+^ or K^+^ currents or the ability of neurons to fire APs. This is important, because it indicates that electrically functional neurons can be derived from LS patient NSCs. However, those neurons manifested a marked dysregulation of calcium homeostasis.

Ca^2+^ is an intracellular signal responsible for controlling numerous cellular processes [27]. The Ca^2+^ signaling network requires first the ON mechanisms, by which there is a 10-fold rise of cytoplasmic Ca^2+^, followed by the OFF mechanisms, which ensure that Ca^2+^ is recovered to basal levels [27]. The increase of cytoplasmic calcium is the consequence of the entry of external Ca^2+^ through different types of channels (VOCs, ROCs, SOCs) and the release of Ca^2+^ from internal stores [27]. At the same time, the OFF mechanisms include the extrusion of calcium to the outside, its return to internal stores (endoplasmic reticulum and mitochondrion) and its association with cytosolic buffers (parvalbumin, calbindin-D28k, calretinin) [27,28]. In the presynaptic membrane of neurons, the rise of cytoplasmic Ca^2+^ triggers the release of neurotransmitters. As important as the rise is the buffering of that calcium to ensure the possibility of a new activation. In fact, sustained high concentrations of cytoplasmic Ca^2+^ are associated with neuronal death [29] through apoptosis if there is ATP available or necrosis if there is ATP depletion [30,31]. It has been postulated that the addition of KCl induces a slow depolarization of membrane potential (∆Ψ), consequent activation of Ca^2+^ voltage-operated channels (VOCs) and Ca^2+^ influx into the cell [32,33]. Cells that respond to this KCl are called evoked cells [32]. The analysis of the response of evoked cells harboring the mtDNA mutation m.13513G>A manifested a slower buffering capacity and an increased refractory period. This is not surprising, because mitochondria play a significant role in shaping global Ca^2+^ signals [33] through direct or indirect participation [34]. The decreased buffering capacity that we observe can lead to higher amounts of calcium in the cytoplasm and consequent neuronal necrosis observed in LS patients [29]. The concrete mechanism by which this mtDNA mutation causes inappropriate buffering of calcium remains unknown. Recently, neural progenitor cells (NPCs) harboring the homoplasmic m.9185T>C mutation in the *MT-ATP6* causative of episodic paralysis with spinal neuropathy (NPC_ATP6) has been reported [35]. In contrast to our results, NPC_ATP6 mutated cells manifested a decreased calcium release (ON mechanisms) rather than the decrease in calcium buffering observed here (OFF mechanism). These differences could be the consequence of the ΔΨmit, in which mutations that affect the ATP synthase increase ΔΨmit and affect calcium release, while mutations in the ETC provoke a diminishment in ΔΨmit and affect calcium buffering. In support of our findings, iPSC-derived neurons from other neurodegenerative diseases such as Parkinson’s disease (PD) or frontotemporal lobar degeneration tauopathy (FTLD-Tau) have also shown an impaired calcium homeostasis as the underlying pathogenic mechanism [36,37]. It is also very interesting that the necrosis observed after myocardial ischemia or stroke [38,39] occurs in response to a lack of oxygen to the mitochondria, suggesting that MD, similarly to PD and TFLD-Tau and other conditions such as myocardial infarction or ischemic stroke, could occur through the same calcium overload process, which leads to necrosis. Although other studies have concluded that mutations in the *MT-ND5* gene in fibroblasts or cybrids can be associated to calcium handling defects [22,40], the phenotype of the neurons, the cell type in which the disease occurs, remained unexplored. Here, we demonstrate defective OXPHOS and a clear dysregulation in calcium homeostasis in neurons harboring the m.13513G>A mutation, with profound consequences for the pathology.

## 4. Materials and Methods

### 4.1. Cell Culture, iPSC Generation and Characterization

This study was approved by the Institutional Ethical Committee of the Autonoma University of Madrid according to Spanish and European Union legislation and complies with the principles of the 1964 Helsinki declaration. Normal human dermal fibroblasts (NHDFs) were purchased from Promocell (C12300) and maintained in Dulbecco’s modified Eagle medium (DMEM) supplemented with 10% FBS, 50 U/mL penicillin and 50 µg/mL streptomycin. Leigh fibroblasts from a described patient harboring the heteroplasmic mutation in the mtDNA m.13513G>A (p.D393N) were kindly provided by Dr. Francina Munell from the Hospital Universitario Vall d’Hebron (Barcelona, Spain), with prior informed consent for study participation. LS fibroblasts were maintained in the same growth medium as NHDF but supplemented with 50 µg/mL uridine. A previously described iPSC line, LND554SV.4 [41], and a control iPSC line reported here (N44SV.1) have been derived, maintained and characterized following the protocols described in [13].

### 4.2. Oxygen Consumption

Oxygen consumption measurements in fibroblasts and iPSCs were performed in an Oroboros Oxygraph-2k and analyzed using DatLab4 software (Oroboros Instruments). Approximately one million fibroblasts or two million iPSCs were used in each chamber. An intact cell measurement protocol has been used. The experimental regime started with routine respiration (Cr), which is defined as respiration in cell-culture medium without additional substrates. After reaching steady-state respiratory flux, ATP synthase was inhibited with oligomycin (2 µg/mL), followed by uncoupling of oxidative phosphorylation by titration of FCCP (carbonyl cyanide p-trifluoromethoxyphenylhydrazone) with 0.5 µM steps (CrU). Finally, respiration was inhibited by sequential addition of rotenone at 0.5 µM (to test for the effect of inhibiting complex I activity) (CRot) and antimycin A at 2.5 µM (for inhibiting complex III) (non-mitochondrial respiration, ROX). Values were normalized by number of cells, and ROX was subtracted. Basal respiration (Cr-ROX), maximum respiration (CrU-ROX), complex I contribution, CrU-(CRot-ROX), or complex II contribution (CRot-ROX) were calculated from a minimum of three independent experiments. Oxygen consumption measurements of induced neurons (iNs) were performed in a Seahorse XFe96 Analyzer and analyzed by Wave software (Agilent Technologies). Induction of iNs from iPSCs was performed in the seahorse microplate following a previously described protocol [14] and measured at day 7, after puromycin selection. Before the experiment, growth medium was replaced by XF-Base Medium (Agilent Technologies) supplemented with 2 mM L-glutamine, 5 mM sodium pyruvate and 10 mM glucose (pH 7.4), and cells were kept 1h at 37 °C at atmospheric O_2_ and CO_2_. Oxygen consumption was determined at basal conditions (Cr) as well as after addition of the following drugs: 500 µM NV241 or DMSO as vehicle, FCCP titration (0.125, 1 or 2 µM), 2 µM rotenone (CRot) and 1 µg/mL antimycin A (ROX). After the experiment, Hoechst 33342 was added to the wells, and pictures were acquired in a fluorescence microscope to quantify DAPI surface. Values were normalized by DAPI surface and non-mitochondrial respiration (ROX) was subtracted. Basal respiration (Cr-ROX), maximum respiration (CrU-ROX), complex I contribution, CrU-(CRot-ROX), or complex II contribution (CRot-ROX) and effect of NV241 were calculated from three independent experiments.

### 4.3. Protein Extraction and Western Blot

Protein was extracted using cold RIPA buffer (50 mM Tris-HCl, pH 7.4, 1% NP-40, 0.5% Na-deoxycholate, 0.1% SDS, 150 mM NaCl, 2 mM EDTA, 50 mM NaF) supplemented with protease inhibitor cocktail (Roche, 11873580001) and quantified using BCA Protein Assay Kit (Thermo Fisher Scientific 23225). Fifty micrograms of protein was separated on a 12% SDS-PAGE gel and electrotransferred to Immobilon-P membranes (Millipore, IPVH00010). Primary antibodies used were Mitoprofile (Abcam ab110411; 1:1000), CS (Abcam ab96600; 1:10000) and GAPDH (Abcam ab8245; 1:6000). Appropriate secondary antibodies coupled to horseradish peroxidase were used, and peroxidase activity was tested using ECL (GE Healthcare, RPN2209). Quantification of WB was performed by densitometric analysis in Image J; values were normalized by total protein amount (GAPDH) and/or by mitochondrial mass (CS).

### 4.4. Lactic Acid Production

Extracellular lactic acid concentration in the cell culture medium was determined using a lactate-dehydrogenase activity assay. First, 500,000 fibroblasts were plated on P100 culture dishes. The next day, growth medium was changed by fresh medium; exactly 24 h after medium was changed, 100 µL was removed, deproteinized and adjusted to pH 6–8. Samples were kept at −80 °C until assay. For the assay, 15 µL of the sample was incubated with 30 µL of NAD+ 15 mM, 5 µL of LDH 1 mg/mL (Roche, 10 127 230 001), 150 µL of assay buffer (consisting of 0.5 M glycine, 0.2 M hydrazine and 3.4 mM EDTA; pH 9.5) and adjusted to a final volume of 300 µL with bidistilled water. This reaction was incubated for 105 min at 37 °C before measuring absorbance at 340 nm. The lactate present in the sample together with NAD^+^ was transformed by lactate dehydrogenase in NADH, with concentration proportional to the increase in absorbance at 340 nm. A standard curve of lactate ranging from 4 to 0.25 mM was used to extrapolate the lactate concentration present in the sample. These values were normalized by the total amount of protein present in the culture measured with BCA Protein Assay Kit (Thermo Scientific, Waltham, MA, USA, Ref 23225). For iPSCs, a similar protocol was used, but starting cell density was 80% confluent. The choice of measurement at 24 h after changing the medium was determined using previous kinetics data. At least three independent experiments were performed.

### 4.5. Respiratory Chain Activity Determination by Spectrophotometry

Measurements of the specific activities (SA) of respiratory chain complexes were determined following the protocol established in [42]. Values were normalized with the specific activity (SA) of citrate synthase (CS). A minimum of three independent experiments was performed.

### 4.6. NSC Generation and Neuronal Differentiation

For generation of NSC, the commercially available PSC Neural Induction Medium (ThermoFisher, Waltham, MA, USA, A1647801) was used, following manufacturer´s instructions. NSCs were routinely maintained in NEM medium (ThermoFisher, Waltham, MA, USA). For neuronal differentiation, 42,000 cells/cm^2^ were seeded in GFR Matrigel-coated plates in NEM medium and maintained for 48 h. After this, growth medium was replaced with differentiation medium consisting of DMEMF12 (Thermo Fisher; Waltham, MA, USA, 11330-057) supplemented with 1x N2 (Thermo Fisher Scientific, Waltham, MA, USA, 1750200), 1x B27 (Thermo Fisher Scientific, Waltham, MA, USA, 17504044), 1x NEAA (Thermo Fisher Scientific, Waltham, MA, USA, 11140035) and 100 μM β-mercaptoethanol (Thermo Fisher Scientific, Waltham, MA, USA, 21985023). Culture medium was changed every other day for 6 weeks. For Patch Clamp and Calcium Imaging experiments, BrainPhys (Stemcell Technologies, Grenoble, France, 05792) was used instead of DMEMF12.

### 4.7. Mutation Analysis and Heteroplasmy Quantification of mtDNA m.13513G>A Mutation

For this purpose, the protocol described in Galera-Monge et al. [41] was followed. Briefly, total DNA was extracted using a standard phenol-chloroform protocol. For mutation analysis, amplification by PCR of a mtDNA region containing the m.13513G>A position was carried out using the following primers: mt-20F: 5′ ATCTGTACCCACGCCTTC 3′ and mt-20R: 5′ AGAGGGGTCAGGGTTGATTC 3′. Following PCR amplification, direct sequencing of amplicons was performed on both strands in an ABI 3730 sequencer (Applied Biosystems, Foster City, CA, USA) using a dye terminator cycle sequencing kit (Applera, Rockville, MD). Heteroplasmy quantification of m.13513G>A mutation was studied by RFLP. PCR amplification with the following primers: 13513F: 5′GACTGACTGACTGACAAGTCAACTAGGACTCATAATA3´ and 13513R: 5′CAGGCGTTTGTGTATGATATGTTTGCGGTTTCGATGACGTGG3´ was followed by digestion with restriction enzyme PflFI (New England Biolabs. Reference: R0595S) and quantified with the Agilent DNA 1000 Kit (Agilent, Santa Clara, CA, USA, 5067-1504) in an Agilent 2100 Bioanalyzer.

### 4.8. Proliferation Assay

Here, 90,000 NSCs were plated onto matrigel-coated 13 mm coverslips in P24 multiwell plates with NEM medium. At 48 h, 10 µM EdU was added to the media and incubated for one hour. After that, cells were washed with PBS, fixed with 4% PFA and permeabilized with 0.025% Triton in PBS. The Click-iT EdU Kit (Invitrogen, Carlsbad, CA, USA, C10338) was used for the detection of EdU, following the manufacturer´s instructions. Hoechst 33342 was added to stain nuclei. Quantification was performed manually at 40× in a randomized and blinded manner (*n* = 5).

### 4.9. Immunofluorescence

Cells were fixed with 4% PFA, permeabilized and blocked with 0.3% Triton X-100 and 3% Donkey Serum in TBS. Samples were incubated with the following primary antibodies overnight at 4 °C: MAP2 (Sigma, St. Louis, MO, USA, M1406,1:250); GFAP (Dako, Carpinteria, CA, USA, Z0334, 1:500); GAD65/67 (Abcam, Branford, CT, USA, ab11070, 1:500); KGA (Abcam, Branford, CT, USA, ab156876, 1:200); TUJ1 (Sigma, St. Louis, MO, USA, T8660, 1:400); MAP2 Chicken (Abcam, Branford, CT, USA, ab5392, 1:10000). Alexa Fluor Dye secondary antibodies were applied (1:500) at room temperature for 30 min. Images were acquired with a spectral confocal microscope LSM710 (Zeiss, Matesalka, Hungary).

### 4.10. iN Morphology Analysis

iPSC-iNs were generated following the protocol described in [14]. Lentiviral GFP+ and unlabeled iNs were mixed in a proportion of 1:6 in order to assess morphology of individual neurons. Images were directly acquired using a BX61 fluorescence microscope (Olympus, Tokyo, Japan).

### 4.11. Electrophysiology

iPSC-derived neurons cultured on coverslips were transferred to the recording chamber and held down with a small piece of platinum wire [43]. The coverslip was constantly perfused with carbonated artificial cerebral spinal fluid (in mM: 119 NaCl, 2.5 KCl, 1.3 MgSO_4_, 2.5 CaCl_2_, 26 NaHCO_3_, 1.25 NaH_2_PO_4_ and 11 glucose; pH ~7.4) at 34 °C. Recording pipettes were filled with intracellular solution (in mM: 122.5 potassium gluconate, 12.5 KCl, 10 HEPES, 0.2 EGTA, 2.0 MgATP, 0.3 Na_2_-GTP, 8.0 NaCl; pH ~7.3) and had a resistance of 4–12 MΩ. Target cells were visualized using a water immersion objective (Olympus, 40×), and whole-cell patch clamp recordings were performed with a HEKA double patch clamp EPC10 amplifier using Patch Master for data acquisition. Voltage and current clamp recordings were used for the electrophysiological characterization. Sodium and potassium currents were evoked by a series of 200 ms long voltage steps (from −70 to +40 mV in 10 mV steps) and inhibited with 1 μM TTX and 10 mM TEA, respectively. Series of current steps (0–200 pA in 10 pA steps) and current ramps from 0–300 pA were performed to determine the cells’ ability to generate action potentials. Data were analyzed offline with FitMaster and IgorPro. At least nine cells of each condition were analyzed.

### 4.12. Calcium Imaging

For calcium imaging, NSCs were allowed to differentiate for 6 weeks (see NSC differentiation section) on Glass-Bottom P35 culture dishes coated with 15 µg/mL Poly-L-Ornithine (Sigma, St. Louis, MO, USA, P3655) at RT for 1 h and 10 µg/mL laminin at 37 °C for 2 h (Thermo Fisher Scientific, Waltham, MA, USA, 23017-015) [43]. The day of the experiment, cells were loaded with 1 ng/µL of Fluo-4 AM (Molecular Probes, Eugene, OR, USA, F14201) diluted in growth medium without Phenol Red and incubated at 37 °C and 5% CO_2_ for 30 min. Before imaging, medium was changed to fresh growth medium without Phenol Red to wash away excess Fluo-4 AM. Time-lapse recordings were acquired using a Zeiss confocal microscope at maximal speed during 30 min. At minute 6, 90 mM KCl was added to the culture plate. Four videos of each condition and independent culture plate were recorded for analysis. For quantification, 10 cells with response to KCl were chosen randomly, and the type of response was analyzed.

### 4.13. Statistical Analysis

Data are presented as mean ± SD. An unpaired two-tailed t-test or Mann–Whitney test was performed; p-values of less than 0.05 were considered statistically significant. (* *p*-value < 0.05; ** *p*-value < 0.01; *** *p*-value < 0.001).

## Supplementary Materials

Supplementary Materials can be found at https://www.mdpi.com/1422-0067/21/9/3191/s1.

## Abbreviations

LS	Leigh syndrome
MD	Mitochondrial disorder
iPSCs	Induced pluripotent stem cells
mtDNA	Mitochondrial DNA
NSCs	Neural stem cells
OXPHOS	Mitochondrial oxidative phosphorylation system
DMSO	Dimethyl sulfoxide
FCCP	Carbonyl cyanide p-trifluoromethoxyphenylhydrazone
RC	Respiratory chain
ROS	Reactive oxygen species
MELAS	Mitochondrial myopathy, encephalopathy, lactic acidosis and stroke
ATP	Adenosine triphosphate
ETC	Electron transfer chain
rRNA	Ribosomal ribonucleic acid
tRNA	Transfer RNA
CS	Citrate synthase
∆Ψmit	Mitochondrial membrane potential
AP	Action potential
FRU	Fluorescence relative units
