# Supplementary material for: Mitochondrial Dysfunction and Calcium Dysregulation in Leigh Syndrome Induced Pluripotent Stem Cell Derived Neurons
Source: Int J Mol Sci. 2020 Apr 30;21(9):3191. doi: 10.3390/ijms21093191 (PMC7247580; doi:10.3390/ijms21093191)
Supplement: Supplementary file 1 [file ijms-21-03191-s001.zip › Supplementary Figure S1.docx]

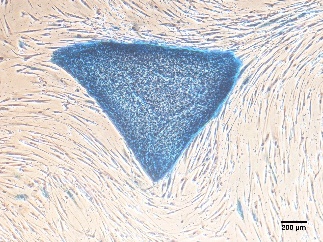

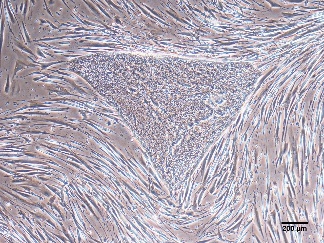

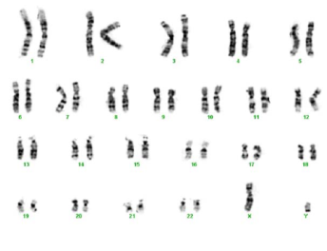

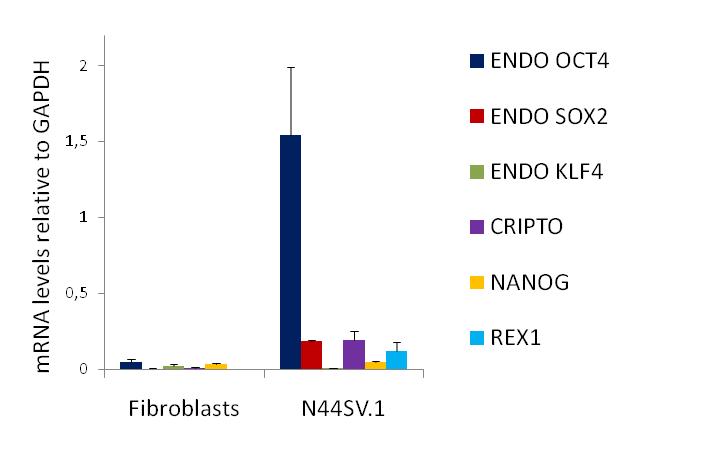

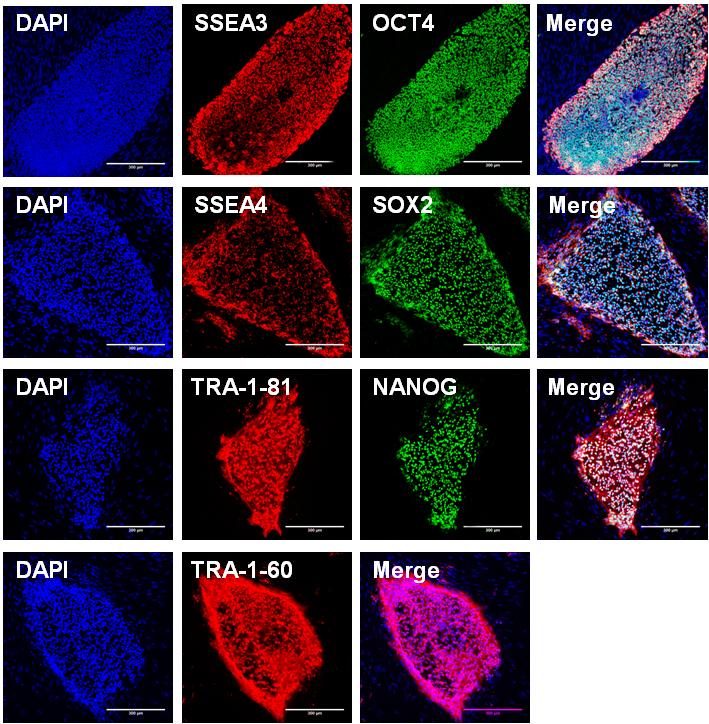

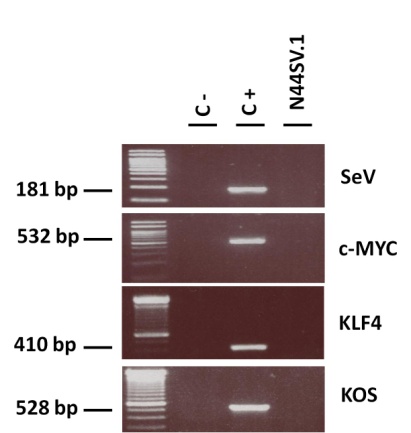

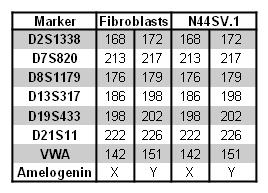

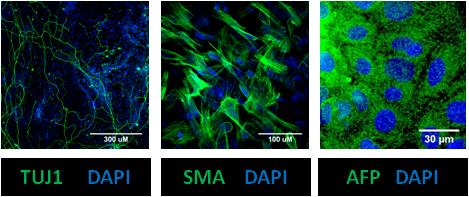


**B**

**C**

**H**

**E**

**G**

**D**

**F**

**I**


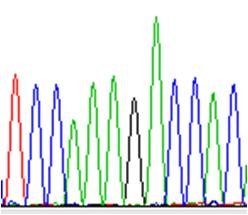


**A**

m.13513G


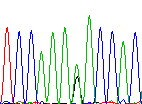


m.13513G>A

Control

Mutant

**Supplementary Figure S1.** Generation and characterization of N44SV.1. **A**) Electropherograms showing the presence of the m.13513G>A mutation in LND554SV.4 and the absence in N44SV.1. **B**) Typical ES-like colony morphology of the N44SV.1 iPSC line growing on feeder cells. **C**) Positive staining for alkaline phosphatase. **D**) qPCR showing the expression of the pluripotency associated genes *OCT4, SOX2, KLF4, CRIPTO, NANOG* and *REX1*. All data normalized with GAPDH. **E**) Immunofluorescence analysis showing expression of typical pluripotent ES cell markers: OCT4, NANOG, SOX2, SSEA3, SSEA4, TRA-1-60 and TRA-1-81; scale bars: 300 µm. **F**) Embryoid body based in vitro differentiation assays demonstrating the ability of N44SV.1 to differentiate into ectoderm (Tuj1+), mesoderm (SMA+), endoderm (AFP+). **G**) RT-PCR for detecting the clearance of the vectors used for reprogramming. **H**) Normal Karyotype (46, XY) in N44SV.1 iPSC line. **i**) DNA fingerprinting analysis from fibroblast and N44SV.1 revealing genetic identity.
